# Validation of dynamic virtual faces for facial affect recognition

**DOI:** 10.1371/journal.pone.0246001

**Published:** 2021-01-25

**Authors:** Patricia Fernández-Sotos, Arturo S. García, Miguel A. Vicente-Querol, Guillermo Lahera, Roberto Rodriguez-Jimenez, Antonio Fernández-Caballero

**Affiliations:** 1 Servicio de Salud Mental, Complejo Hospitalario Universitario de Albacete, Albacete, Spain; 2 Biomedical Research Networking Center in Mental Health (CIBERSAM), Madrid, Spain; 3 Instituto de Investigación en Informática de Albacete, Albacete, Spain; 4 Departamento de Sistemas Informáticos, Universidad de Castilla-La Mancha, Albacete, Spain; 5 Departamento de Medicina y Especialidades Médicas, Universidad de Alcalá, Madrid, Spain; 6 Instituto de Investigación Sanitaria Hospital 12 de Octubre (imas12), Madrid, Spain; 7 CogPsy-Group, Universidad Complutense de Madrid, Madrid, Spain; University Hospitals Tubingen: Universitatsklinikum Tubingen, GERMANY

## Abstract

The ability to recognise facial emotions is essential for successful social interaction. The most common stimuli used when evaluating this ability are photographs. Although these stimuli have proved to be valid, they do not offer the level of realism that virtual humans have achieved. The objective of the present paper is the validation of a new set of dynamic virtual faces (DVFs) that mimic the six basic emotions plus the neutral expression. The faces are prepared to be observed with low and high dynamism, and from front and side views. For this purpose, 204 healthy participants, stratified by gender, age and education level, were recruited for assessing their facial affect recognition with the set of DVFs. The accuracy in responses was compared with the already validated Penn Emotion Recognition Test (ER-40). The results showed that DVFs were as valid as standardised natural faces for accurately recreating human-like facial expressions. The overall accuracy in the identification of emotions was higher for the DVFs (88.25%) than for the ER-40 faces (82.60%). The percentage of hits of each DVF emotion was high, especially for neutral expression and happiness emotion. No statistically significant differences were discovered regarding gender. Nor were significant differences found between younger adults and adults over 60 years. Moreover, there is an increase of hits for avatar faces showing a greater dynamism, as well as front views of the DVFs compared to their profile presentations. DVFs are as valid as standardised natural faces for accurately recreating human-like facial expressions of emotions.

## Introduction

The ability to identify emotions on others’ faces is crucial for effective social interaction [1–3]. Emotional facial expressions convey relevant information about others and oneself and serve to regulate the environment, indicating people’s intentions and behaviours [4, 5]. Thus, the way an individual recognises the emotional state in the other determines a large part of social success, which is relevant to his/her functioning in the community [6–8].

There is consistent evidence that patients with different neuropsychiatric conditions such as autism [9, 10], schizophrenia [11, 12], major depressive disorder [13], bipolar disorder [14, 15], Alzheimer’s dementia [16], or Prader-Willi syndrome [17] have difficulty in accurately recognising the emotions expressed by others. This deficit can generate a misinterpretation of social situations and, therefore, a significant deficit in social functioning and quality of life [18]. Due to the relevance of facial recognition of emotions on social functioning, today, there is a large amount of research focused on the design and validation of tools that assess the ability to recognise emotions.

In most emotional recognition studies, the experimental stimulus is presented through photographs or static images [19, 20]. Some authors point out that this stimulus does not reflect the reality of the facial stimulus [21]. Other studies use videos to present expressions in a genuine way [22–24]. However, video tasks have not been properly validated and have several limitations in their format [23]. In turn, the main limitation is when they have to be integrated into a simulated interaction [25].

Given the adaptive value of the adequate prediction of emotions for survival, emotional recognition by faces is probably one of the brain’s most specific skills and evolutionary refinement in human development. As it is known, facial affect recognition is based on subtle and precise information from the stimulus. Thus, the presentation of a dynamic facial expression generates a more intense emotional experience and facilitates successful emotional recognition [26–29]. In this sense, virtual reality is a powerful tool that provides environments and situations similar to reality, dynamic avatars that allow social interaction with the participant, and that can be managed to represent different emotional states [30, 31]. The ecological validity of this approach lies in the precise presentation and control of the dynamic perception of stimuli. VR allows combining the real control of laboratory measures with the verisimilitude of everyday experiences [32]. The development of realistic virtual humans appears to be one of the most important challenges to achieve successful interaction and to simulate the complex reality of mental processes and human social behaviour through technology [33].

Most of the studies published in relation to the creation of avatar faces take as reference the Facial Action Coding System (FACS) developed by Ekman and Friesen [34]. The basic element of this system is the action unit (AU), which represents muscular activity. This produces momentary changes in facial appearance so that expressions are encoded by detecting the presence of combinations of AUs in the face. First studies used black and white virtual faces, without paying attention to the details of the face, comparing directly with the Pictures of Facial Affect (POFA) [35, 36]. Emotion recognition was effective, despite the lack of realism of the faces [37, 38]. Subsequently, a new study validated a set of virtual facial stimuli without dynamism, comparing it with natural emotions shown in photographs that had previously been validated [39, 40], concluding that virtual faces were as valid as natural images [41]. In 2012, a new set of dynamic avatars called FACSGen 2.0, was developed. It included black and white faces representing eight emotions (anger, disgust, embarrassment, fear, happiness, pride, sadness, and surprise) with two levels intensity [31]. Virtual faces were compared with a validated set of filmed emotion expressions, the Amsterdam Dynamic Facial Expression Set (ADFES) [42], obtaining high rates of emotional recognition for all the emotions evaluated, with results similar to those obtained with real faces. Gutiérrez-Maldonado and collaborators designed a new set of avatars, which represent five basic emotions (anger, disgust, fear, happiness and sadness), with two levels of intensity [25]. The virtual stimulus was compared with a natural stimulus selected from the Penn Emotion Recognition Test-96 Faces version (PERT96) [40]. Repeated-measures ANOVA revealed no significant difference in participants’ accuracy of recognition between the two presentation conditions.

In our view, no facial stimulus published previously includes some characteristics that may be relevant when studying different aspects of emotion recognition, such as the degree of dynamism of the neck and shoulders or the different locations that people can have in a social interaction (front position, laterally position). In turn, most sets of dynamic virtual faces (DVFs) included adult white male and female avatars. For this reason, our team has designed a new set of DVFs that can be presented from different angles, including front angle, right and left profile. Moreover, the avatars have two degrees of dynamism (high and low). Less dynamism involves movements of the face only, while greater dynamism incorporates movement of the neck and shoulders. The set of DVFs included 2 white avatars (female and male) around 30 years old, 2 African avatars (female and male), about 30 years old and 2 avatars of old age (female and male). The complete design process of our new series of DVFs representing the six basic emotions from the neutral expression using AUs has recently been approached from an engineering point of view [30].

Previous research has explored the role of gender, age and educational level in the recognition of facial affect. In general, women show more accurate and rapid recognition of facial emotions compared to men [43, 44]. Explanations for the sex difference range from sexual inequalities in power and social status [45] to evolutionary perspectives based on the almost universal responsibility of women in sex parenting [46]. However, this apparent superiority has been objectified mainly when exposed to stimuli with static facial expressions and / or full intensity. With regard to age, recent studies indicate that facial affect recognition from the age over 60 worsens with respect to younger adulthood [47]. This would suggest that older adults pay less attention to socially relevant areas such as eyes or mouth [48]. Another study identified a correlation between years of education and identification of the six basic emotions [49]. These results are consistent with previously published works that support the general role of education in predicting cognitive performance in multiple neuropsychological tests [50].

The main objective of this work was to validate a new set of highly realistic DVFs that could subsequently be integrated into a social cognition training therapy for patients with schizophrenia and related disorders. The precision in the responses provided by a large stratified sample of healthy controls to the DVF pool was compared with the precision in the responses to the validated Penn emotion recognition test (ER-40) [15, 40]. The following hypotheses were established:

- H1. The number of hits (correct emotion identifications) and the reaction time (time calculated from the appearance of the stimulus to the participant’s response) of the set of dynamic virtual faces (DVFs) will be better than the face photos of the ER-40.
- H2. Within the set of DVFs, the participants will recognise more precisely the most dynamic as opposed to the less dynamic ones, obtaining a greater number of hits and a shorter reaction time.
- H3. Within the set of DVFs, the participants will recognise more accurately the faces presented in front view compared to the faces oriented laterally, obtaining a greater number of hits and shorter reaction time in frontal views.
- H4. Within the set of DVFs, there will be differences in the number of hits and reaction time regarding gender (in favour of women), age (in favour of participants younger than 60 years) and education level (in favour of higher educational levels)

## Materials and methods

### Participants

The study was carried out with healthy volunteers. The single inclusion criterion was that the participants had to be between 20 and 79 years old. Exclusion criteria included a diagnosis of mental illness, a personal history of medical illness (that could interfere with affect recognition), and a first-degree family history of psychosis. The sample was stratified by gender (50% men, 50% women), age (divided into 3 age ranges: 20–39, 40–59, 60–79), and education level (divided into the three educational strata; ≤ 2, basic level; 3–4, medium level; ≥ 5, high level). The stratification lead to the conclusion that an exact number of 204 participants had to be enrolled. Indeed, Table 1 was prepared considering the level of education of the Spanish population in 2017. 43% of men had basic level studies compared to 38.3% of women; 22.7% of men and women had medium level studies; 33.8% of men had high level studies compared to 38.9% of women.

**Table 1:** Number of participants stratified by gender, age and education level.

	Age and gender					
	20–39		40–59		50–79	
	Men	Women	Men	Women	Men	Women
**Basic education level (0–2)**						
Men = 45 (43.5%); Women = 39 (38.3%)	15	13	15	13	15	13
**Medium education level (3–4)**						
Men = 24 (22.7%); Women = 24 (22.7%)	8	8	8	8	8	8
**High education level (5–8)**						
Men = 33 (33.8%); Women = 39 (38.9%)	11	13	11	13	11	13

Note: Total number of participants = 204.

### Procedure

A data collection notebook was designed by the research team to annotate sociodemographic and clinical data. The sociodemographic data included age, gender and level of education, among others. The clinical data included personal somatic history (including neurological), toxic personal history, psychiatric personal history, and relevant family psychiatric history. The Mini International Neuropsychiatric Interview [51], Spanish version 5.0.0, was used for screening of psychiatric disorders. The Spanish version [52] of the Positive and Negative Affect Schedule (PANAS) [53] was also administered with the aim to exclude participants with altered mood state in the moment of performing the emotion recognition task. PANAS is a 20-item self-report questionnaire which measures an individual’s positive and negative affect. If a participant had a positive affect score of less than 25 (PA < 25) or a negative affect score of more than 35 (NA > 35), he/she was excluded from the study, in line with a previous work [32].

All participants read and signed a written consent before starting their session. Data collection was carried out in a single 60-minute individual session. The facial stimuli were presented in a random order in 2 separate blocks (classic Penn Emotion Recognition Test (ER-40) already validated versus the DVFs created by the research team). In the presentation block of the DVFs, the order of appearance of the avatar faces was also randomised for each participant.

The two main measures for each face presented are the hits and the response or reaction time. The reaction time for ER-40 is determined from the appearance of the emotion photograph. In the case of DVFs, as a transition is made from the neutral expression to the target emotion (and back to neutral), it makes no sense for the participant to answer before the transition is initiated, so the reaction time is counted from that precise moment. The study was approved by the Clinical Research Ethics Committee.

### Novel dynamic virtual faces

All participants were shown 52 DVFs on a 27-inches computer screen. They had to identify all basic emotions presented (happiness, sadness, anger, fear, disgust and surprise) plus the neutral expression. Each exhibited emotion started from and ended in the neutral expression, with a total presentation time of 2 seconds. Different virtual characters were selected from the ones available in Adobe Fuse CC [54], a character generator tool commonly used by game developers. These characters were enhanced with new blendshape animations reproducing a set of AUs that allowed us to create realistic emotion animations based on Ekman’s work. The technical details of the implementation of the virtual faces are available in [55, 56]. The software tool developed for this research is publicly accessible at Universidad de Castilla-La Mancha institutional repository RUIdeRA at hdl.handle.net/10578/27021. This repository entry also contains the raw data files used in the statistical analysis described in this paper.

The participants had to identify each emotion expressed by a DVF among the seven options offered. Of these 52 faces, 50% were interspersed with less dynamism (the movement zone includes only the most characteristic facial features of each emotion) and 50% revealed more dynamic faces (the movement zone added movements of the neck and shoulders that should bring more realism and naturalness to the scene). Moreover, both the most and least dynamic faces were shown 50% in frontal view, 25% in right side face and 25% in left side face. A video demonstrating the two levels of dynamism and several views of a same DVF is available as Supporting information (see S1 Video).

The set of DVFs included 2 white avatars around 30 years old, holding different features in terms of eye colour, skin tone and hair. Additionally, 2 avatars were designed of the African race, about 30 years old and 2 avatars of old age. Of the 52 avatars presented, 8 were of African race and 8 were of old age.

### Classical Penn Emotion Recognition Task

The Penn Emotion Recognition Task (ER-40) [39] contains forty colour photographs of faces of different ethnicity, expressing the four basic feelings happy, sad, angry and fearful, as well as the no emotion expression. It includes eight photographs of each expression (four high-intensity and four low-intensity ones). This test has strong psychometric properties and has been identified by some authors as recommended when used in clinical trials [57].

### Statistical analysis

IBM SPSS Statistics (version 24) was used to conduct the statistical analyses. Since the data (hits and reaction time) did not follow a normal distribution, mainly non-parametric tests were used for hypothesis testing. A *p*-value < .05 was considered to be statistically significant.

Regarding the statistical tests, the Kruskal-Wallis test was used to determine if there were statistically significant differences in the number of correct answers (hits) and reaction time between more than two different groups of participants (the case of age and education groups). In the cases in which this test found differences, the Dunn’s post-hoc test together with a Bonferroni correction for pair-wise comparisons was applied in order to find out which groups was different. When only two groups were compared, the Mann-Whitney test was used instead. Finally, in the cases in which we wanted to find differences in the performance of the same group of participants using two techniques (i.e. DVF with lower versus higher dynamism), the Wilcoxon Signed Ranks test was used.

## Results

As mentioned before, non-parametric tests were used to compare the results since the number of correct answers did not follow a normal distribution (Kolmogorov-Smirnov test: Z = 0.165, p < .001).

### Accuracy in emotional expression recognition

With regards to the accuracy in emotional expression recognition using the ER-40 image dataset, the percentage of successful recognition is high (82.6%) as shown in Table 2. The no emotion expression has the lowest recognition percentage (70.6%) and fearful the highest one (94.0%).

**Table 2:** Emotion recognition confusion matrix for each category of emotion included in the ER-40 dataset.

	No emotion	Fearful	Angry	Happy	Sad
No emotion	**70.6%**	2.5%	8.3%	18.3%	0.4%
Fearful	3.2%	**94.0%**	0.9%	0.9%	1.1%
Angry	3.0%	14.0%	**78.6%**	4.1%	0.3%
Happy	1.1%	10.4%	4.1%	**81.9%**	2.5%
Sad	0.1%	1.2%	1.0%	9.8%	**87,9%**

Note: Columns: emotions recognised. Rows: emotions presented.

In respect to the DVFs, as shown in Table 3, the percentage of successful recognition for each DVF expressing an emotion is even higher, all above 85% except for fear. A closer look shows that fear was mainly confused with surprise and sadness. Regarding the rest of the emotions, the only one with a quite high confusion percentage is disgust, which was mainly confused with anger. Apart from neutral expression, happiness is the emotion that obtained a higher recognition percentage.

**Table 3:** Emotion recognition confusion matrix for each emotion depicted by DVFs.

	Neutral	Surprise	Fear	Anger	Disgust	Happiness	Sadness
Neutral	**96.3%**	0.4%	0.1%	0.2%	0.5%	0.0%	2.5%
Surprise	0.5%	**91.3%**	7.7%	0.0%	0.2%	0.1%	0.2%
Fear	0.9%	14.4%	**77.0%**	0.2%	0.7%	0.0%	6.8%
Anger	1.0%	1.4%	1.8%	**90.3%**	4.2%	0.1%	1.2%
Disgust	0.9%	1.1%	1.2%	10.6%	**85.8%**	0.0%	0.4%
Happiness	2.8%	0.7%	0.2%	0.4%	0.2%	**95.6%**	0.1%
Sadness	3.6%	3.5%	4.4%	0.6%	2.5%	0.1%	**85.5%**

Note: Columns: emotions recognised. Rows: emotions presented.

### H1. On the presentation of ER-40 static photos and dynamic virtual faces

Regarding the number of hits, the DVFs obtained better results than the ER-40 faces (88.2% and 82.6%, respectively), although participants had a greater number of emotional options with the DVFs compared to the natural ones (7 versus 5). The Wilcoxon Signed Ranks test (Z = -9.145, p < .001) confirmed that the difference in the number of hits is statistically significant. All emotions were better recognised with DVFs except for fear and sadness.

With regards to the reaction time, there was a significant difference in the average reaction time per participant (Wilcoxon Signed Ranks test: Z = -8.965, p < .001). The reaction time using ER-40 was significantly lower than the reaction time using DVFs (M = 2.05 s, SD = 0.78, for ER-40; M = 2.43 s, SD = 0.85 for DVFs).

**Fig 1:**
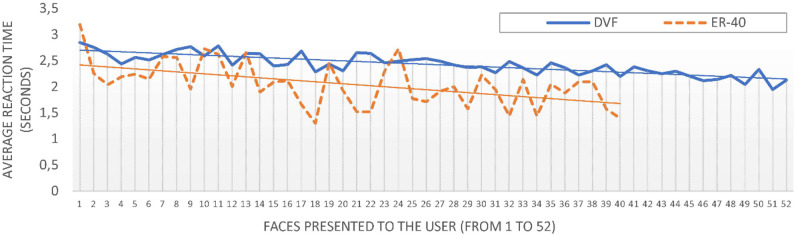
Average reaction time for each face presented to the participants using DVFs (in blue) and the ER-40 dataset (in orange). The figure plots in the X axis the faces to be identified (from 1 to 52 for the DVFs, and from 1 to 40 for ER-40) for the participants. The lines represent the trend of the graphs.

Fig 1 shows the average reaction time of the participants during the progression of the test for the DVF and ER-40 conditions. It also visualises the difference in the reaction time in both parts of the study. The slopes of the trend lines displayed in Fig 1 are -0.011 and -0.019 for the DVF and ER-40 conditions, respectively, which in both cases implies a slight reduction in the reaction time during the progression of the test.

### H2. On the dynamism of the dynamic virtual faces

The confusion matrices for both levels of dynamism are shown in Table 4. The average successful identification percentage was 90.14% for more dynamic DVFs compared to 85.05% for less dynamic ones. Using the less dynamic DVFs, the percentage of confusion was 11.7%, while it was reduced to 2% when more dynamic DVFs were reproduced. All emotions were better recognised with more dynamic DVFs, being the most obvious improvements for disgust (15%) and fear (8.2%). The Wilcoxon Signed Ranks test (Z = -5.666, p < .001) confirmed the hypothesis. A further study of the impact on the individual emotions showed that the increase in the success recognition rate was significant for disgust and sadness (Z = -2.563, p = .010 and Z = -3.399, p = .001, respectively).

**Table 4:** Emotion recognition confusion matrix using high and low dynamism in the DVFs when expressing the emotions.

**High dynamism**						
	Surprise	Fear	Anger	Disgust	Happiness	Sadness
Surprise	**91.9%**	7.1%	0.0%	0.1%	0.0%	0.4%
Fear	15.4%	**81.1%**	0.2%	0.6%	0.0%	2.0%
Anger	1.3%	1.8%	**91.5%**	2.9%	0.1%	0.7%
Disgust	1.1%	1.8%	2.9%	**93.5%**	0.0%	0.4%
Happiness	0.8%	0.4%	0.4%	0.0%	**95.9%**	0.0%
Sadness	4.3%	2.7%	0.8%	2.2%	0.0%	**87.1%**
**Low dynamism**						
	Surprise	Fear	Anger	Disgust	Happiness	Sadness
Surprise	**90.7%**	8.3%	0.0%	0.2%	0.1%	0.1%
Fear	13.4%	**72.9%**	0.1%	0.9%	0.0%	11.7%
Anger	1.5%	1.7%	**89.1%**	5.6%	0.0%	1.6%
Disgust	1.1%	0.7%	17.9%	**78.5%**	0.0%	0.5%
Happiness	0.6%	0.0%	0.5%	0.5%	**95.4%**	0.1%
Sadness	2.6%	6.3%	0.3%	2.7%	0.3%	**83.7%**

Notes: Columns: emotions recognised. Rows: emotions presented. The average successful recognition percentages are 90.14% and 85.05% for higher and lower dynamism, respectively.

Regarding the reaction time, there was a significant difference in the average reaction time depending on the level of dynamism of the DVFs (Wilcoxon Signed Ranks test: Z = -5.973, p < .001). The average reaction time using the most dynamic DVFs (M = 2.34 s, SD = 0.84) was significantly lower than the one obtained using the least dynamic ones (M = 2.50 s, SD = 0.89).

### H3. On the orientation of the dynamic virtual faces

Three different orientations (angles) were randomly used to present the DVFs (50% frontal, 25% right profile and 25% left profile). A significant difference was perceived in the total number of correct answers (Wilcoxon Signed Ranks test: Z = -2.829, p = .005). Focusing on individual emotion identification, the test revealed that there was a significant difference for fear and sadness (Z = -1.996, p = .046 and Z = -3.605, p = .001) in favour of the frontal faces’ presentation. We did not find significant results for the other emotions. The average successful identification percentage for frontal views was 89.63%, compared to 88.02% for lateral views (see Table 5). Although not statistically significant, a better recognition of disgust and anger in profile DVFs is observed. As shown in the table, these two emotions present a higher success rate in the profile condition, in part because they are less confused with each other.

**Table 5:** Emotion recognition confusion matrix using frontal and profile views of the DVFs.

**Frontal views**							
	Neutral	Surprise	Fear	Anger	Disgust	Happiness	Sadness
Neutral	**97.1%**	0.7%	0.0%	0.0%	0.0%	0.0%	2.2%
Surprise	0.6%	**92.4%**	6.6%	0.0%	0.1%	0.0%	0.2%
Fear	0.9%	12.4%	**78.9%**	0.4%	0.9%	0.0%	6.6%
Anger	1.0%	1.5%	2.0%	−**89.7%**	4.5%	0.0%	1.3%
Disgust	0.9%	0.9%	1.6%	11.1%	**85.0%**	0.0%	0.6%
Happiness	3.0%	0.4%	0.0%	0.4%	0.2%	**96.1%**	0.0%
Sadness	2.4%	2.3%	3.9%	0.5%	2.3%	0.2%	**88.3%**
**Profile views**							
	Neutral	Surprise	Fear	Anger	Disgust	Happiness	Sadness
Neutral	**95.6%**	0.0%	0.2%	0.5%	1.0%	0.0%	2.7%
Surprise	0.4%	**90.2%**	8.8%	0.0%	0.2%	0.1%	0.2%
Fear	1.0%	16.4%	**75.1%**	0.0%	0.6%	0.0%	7.0%
Anger	1.1%	1.3%	1.6%	**90.9%**	3.9%	0.1%	1.0%
Disgust	0.9%	1.3%	0.9%	10.1%	**86.6%**	0.0%	0.2%
Happiness	2.6%	1.0%	0.4%	0.5%	0.2%	**95.2%**	0.1%
Sadness	4.7%	4.7%	4.8%	0.6%	2.6%	0.0%	**82.6%**

Notes: Columns: emotions recognized. Rows: emotions presented. Average successful recognition percentages are 89.63% and 88.02% for frontal and profile views, respectively.

### H4. On the issues related to gender, age and education level of the dynamic virtual faces

The Mann-Whitney test did not find any significant difference in the total number of correct answers by gender (U = 5085, p < .781, with M = 45.78, SD = 5.47 for women, and M = 46.01, SD = 4.58 for men). After having looked at the individual emotions, the only significant difference was with disgust (U = 4325, p = .029), which was better identified by women (M = 7.06, SD = 1.15 for women, and M = 6.66, SD = 1.37 for men). No difference was detected regarding reaction time. The average reaction time for women was M = 2.49 s, SD = 0.87, and M = 2.37 s, SD = 0.82 for men.

Regarding age groups, the Kruskal-Wallis test observed significant differences in the total number of hits ($\chi_{\left(2\right)}^{2}=9.659$, p = 0.008). There was significant difference between age groups 40–59 and 20–39 (p = .002). The number of correct answers for age group 20–39 (M = 47.21, SD = 4.08) was significantly higher than the number of hits for age group 40–59 (M = 44.69, SD = 5.43). No more significant differences became aware for any other combination. Focusing on individual emotions, significant differences were found for anger ($\chi_{\left(2\right)}^{2}=6.574$, p = .037) and disgust ($\chi_{\left(2\right)}^{2}=7.463$, p = .024). In both cases, the differences coincide with the study of the total number of correct answers. The results for age group 20–39 (M = 7.54, SD = 1.06 for anger and M = 7.12, SD = 1.02 for disgust) were significantly better than those for age group 40–59 (M = 7.13, SD = 1.35 for anger and M = 6.50, SD = 1.46 for disgust) (p = .015 and p = .011, respectively).

Moreover, we studied the reaction time in the different age groups. The Kruskal-Wallis test revealed significant differences ($\chi_{\left(2\right)}^{2}=34.014$, p < .001). There was a significant difference between age groups 20–39 and 40–59 (p < .001 with M = 1.99 s, SD = 0.76 and M = 2.51 s, SD = 0.82, respectively), and 20–39 and over 60 (p < .001 with M = 2.78 s, SD = 0.78 for over 60), meaning that the reaction time was significantly lower for age group 20–39 than for age groups 40–59 and over 60.

Regarding education levels, significant differences were discovered in the total number of hits ($\chi_{\left(2\right)}^{2}=10.435$, p = .005). A post-hoc pairwise comparison revealed significant differences between basic (0–2) and medium (3–4) education levels (p = .016, with M = 45.49, SD = 5.50 and M = 47.29, SD = 4.99, respectively), and high (5–8) and medium levels (p = .002, with M = 44.38, SD = 4.36 for the high level). The number of hits for medium education level was significantly higher than the number of correct answers for the other education levels. An in-depth study of individual emotions revealed that there was a significant difference between education levels for surprise ($\chi_{\left(2\right)}^{2}=13.515$, p = .001), disgust ($\chi_{\left(2\right)}^{2}=10.200$, p = .006) and sadness ($\chi_{\left(2\right)}^{2}=6.685$, p = .035). For surprise, high education level obtained better results than basic education level (p = .001, with M = 7.60, SD = 0.66 and M = 7.04, SD = 1.08, respectively), while for disgust and sadness medium education level obtained better results than high education level (p = .001 with M = 7.31, SD = 0.96 and M = 6.56, SD = 1.37 for disgust, and p = .010 with M = 7.22, SD = 1.34 and M = 6.56, SD = 1.75 for sadness).

## Discussion

The main objective of this work was to verify whether the emotions expressed by DVFs could be recognised as well as natural emotions in photographs of human faces. As we firstly hypothesised, the results confirmed that DVFs are as valid as photographs of the ER-40 for assessing emotion recognition skills. In fact, not only was the accuracy similar between DVFs and the ER-40 faces, but a statistically significant difference was obtained in favour of DVFs.

The percentage of general success was higher for the condition of DVFs compared to the natural ER-40 faces. For the natural photo faces, the mean of hits was similar to that obtained in previous studies [58]. All emotions were better recognised with DVFs, except for fear and sadness. The reason why fear emotion is recognised worse on DVFs may be that there is no surprised condition in ER-40.

Precisely, it is the one that generates a good piece of the mistakes done when using DVFs. Adding the percentage of answers for surprise to fear when using DVFs would raise the recognition percentage of fear to 91.4%, very close to 94% obtained in ER-40 for fearful. The difference in the number of hits around sadness in the two conditions is not as striking as for fear (2.4%).

The overall accuracy in emotion identification with our DVFs was consistent with similar studies using virtual faces [37, 40, 59, 60]. In this sense, neutral expression and happiness emotion were the most easily recognised, followed by anger and surprise. In our study, although fear was the worst identified emotion, it obtained a percentage of success like previous studies [25, 31].

In line with previous literature, fear was mainly confused with surprise and sadness [38, 41]. This is striking as it differs from the result of our previous study using the same facial expressions in a pilot study [60]. The difference could probably be explained as two emotional expressions (disgust and sadness) were redesigned following the results obtained in the previous study. The recognition of disgust improved from 69.6% to 85.8%, and sadness from 62% to 85.5%. To sum up, the average percentage of successful recognition using this new set of DVFs is very high (88.2%) in comparison to our previously reported high result (83.6%) [41].

For the case of disgust, our results differ from those previously published by other groups. While most previous works have reported a worse recognition rate of disgust compared to other emotions [27, 29, 38, 41], in our study the high success rate is striking. This is probably due to the redesign of the emotion that our team carried out from previous results [60], considering the difficulty of authenticity to recreate the nasolabial area [37].

Contrarily to what was hypothesised, the reaction time using ER-40 is significantly lower compared to DVFs. The most plausible explanation for this result is that the presentation of each emotion began and ended with the neutral expression in the DVF condition, with a total presentation speed of 2 seconds. It is worth noting that, in both cases, the participant could click on an option right after the face was presented, even if it was in the middle of the animation for DVFs. However, the participants normally waited for the emotional transition to end before answering, something that did not happen for ER-40, as there was no transition at all. Another possible explanation for this result is that the number of choices was lower in the ER-40 study. Thus, the participants had less options to consider before responding.

The second hypothesis was confirmed. The most dynamic virtual faces were recognised better and faster than the less dynamic. It makes sense that a greater dynamism in the area of the neck and face is related to a better identification of emotions due to a notably increasing realism. As proposed by previous authors, a dynamic presentation of emotional facial expressions can evoke a better subjective emotional experience [29].

Following our third hypotheses, we could only confirm a significant improvement in the recognition of emotions with frontal views compared to lateral ones for fear and sadness. No significant differences were detected in the reaction time using the different orientations. As far as we know, this is the first work that has studied the effect that the face orientation has on the recognition of emotions. In real-life situations, facial expressions are not always presented frontally. Working with increasingly realistic stimuli will help assessing and remediating the identification of emotions in the everyday environment.

In relation to the fourth hypothesis, we found significant gender differences in emotion recognition in relation to the number of hits for disgust in favour of women, but not for the other emotions. Although it is widely believed that females outperform males in the ability to recognise other people’s emotions, this conclusion seems not well supported by the extant literature. A recent study provides evidence for the presence of gender differences in emotion recognition ability but show that these differences are modest in magnitude and appear to be limited to facial disgust [61], what is congruent with our results. Indeed, some meta-analyses and literature reviews have reported that females outperform males in facial emotion recognition [62, 63] but small effect sizes are generally reported. Regarding age, a significant difference was found between age groups 40–59 and 20–39, with the number of correct answers being higher for the younger group. In addition, a significant difference was found between the age groups 20–39 and 40–59 and 20–39 and over 60, in favour of the younger age group in both cases. Although previous studies indicate that there seems to be an advantage of young adults over older adults in facial affect recognition [47, 64], it should be noted that the fact that the tool was presented on a computer and that participants had to use a mouse to do the task, probably influenced the worst results of the older age groups. Unlike the results obtained in other studies, in reference to educational level, the number of correct answers for the intermediate level of education was significantly higher than the number of correct answers for the other education levels.

The present study has some limitations and strengths. Among the limitations, it should be mentioned that the ER-40 test incorporates only four emotions plus neutral, whereas our DVFs have six emotions plus the neutral expression. But the main objective was not to study differences between accuracy in the two tasks, but to study if the set of created DVFs represented adequately the emotions and that they would be recognised by the participants, as well as the ER-40 photographs of real faces. Perhaps, the strongest aspects of our study are the size and, especially, the stratification of the sample by gender, age and education level. As far as we know, this is the first study with DVFs designed for emotional recognition validated in a representative sample of the general population. Other strengths are related to some the evaluated aspects, such as the orientation view and the level of dynamism of the DVFs.

In conclusion, the present study showed that the set of DVFs was as valid as the standardised natural faces for accurately recreating human-like facial expression. The percentage of correct answers for each DVF emotion was high, especially for neutral expression and happiness. This work provides novel information on the importance of parameters such as the degree of dynamism of the avatars and their locations with respect to the subject evaluated.

## Supporting information

S1 Video Video demonstrating the two levels of dynamism and several views of a same DVF. (MP4)
